# Epidemiology of childhood conduct problems in Brazil: systematic review and meta-analysis

**DOI:** 10.1007/s00127-013-0695-x

**Published:** 2013-05-05

**Authors:** Joseph Murray, Luciana Anselmi, Erika Alejandra Giraldo Gallo, Bacy Fleitlich-Bilyk, Isabel A. Bordin

**Affiliations:** 1 Department of Psychiatry, University of Cambridge, Douglas House, 18b Trumpington Road, Cambridge, CB2 8AH UK; 2 Postgraduate Program in Epidemiology, Universidade Federal de Pelotas, Pelotas, Brazil; 3 Department of Psychiatry, Universidade de São Paulo, São Paulo, Brazil; 4 Department of Psychiatry, Universidade Federal de São Paulo, Pelotas, Brazil

**Keywords:** Conduct disorder, Oppositional defiant disorder, Risk factors, Middle income country, Systematic review

## Abstract

**Purpose:**

This study aimed to review evidence on the prevalence of and risk factors for conduct problems in Brazil.

**Methods:**

We searched electronic databases and contacted Brazilian researchers up to 05/2012. Studies were included in the review if they reported the prevalence of or risk factors for conduct problems, conduct disorder, or oppositional defiant disorder for 100 + Brazilian children aged ≤18 years, systematically sampled in schools or the community. Prevalence rates and sex differences were meta-analysed. Risk factor studies were reviewed one by one.

**Results:**

The average prevalence of conduct problems in screening questionnaires was 20.8 %, and the average prevalence of conduct disorder/oppositional defiant disorder was 4.1 %. There was systematic variation in the results of screening studies according to methodology: recruitment location, informants, instruments, impairment criterion for case definition, and response rates. Risk factors previously identified in high-income countries were mainly replicated in Brazil, including comorbid mental health problems, educational failure, low religiosity, harsh physical punishment and abuse, parental mental health problems, single parent family, and low socioeconomic status. However, boys did not always have higher risk for conduct problems than girls.

**Conclusions:**

Studies using screening questionnaires suggest that Brazilian children have higher rates of conduct problems than children in other countries, but diagnostic studies do not show this difference. Risk factors in Brazil were similar to those in high-income countries, apart from child sex. Future research should investigate developmental patterns of antisocial behaviour, employ a variety of research designs to identify causal risk mechanisms, and examine a broader range of risk factors.

## Introduction

Conduct problems refer to antisocial behaviours that are characteristic of conduct disorder (CD) and oppositional defiant disorder (ODD). CD consists of a repetitive and persistent pattern of behaviours in which the basic rights of others and major age-appropriate societal norms or rules are violated, and ODD refers to a recurrent pattern of negative, hostile, and defiant behaviour with social or educational impairment [1]. CD and ODD are some of the most common child mental disorders found in community settings: among children aged 5–15 years in a national British survey, 1.6 % had CD, 2.3 % had ODD, 0.9 % had a depressive disorder, 3.8 % had an anxiety disorder, and 2.2 % had attention deficit/hyperactivity disorder [2].

According to prominent life-course theories, childhood conduct problems play a critical role in the development of later criminal behaviour and violence [3, 4]. Retrospectively, most antisocial adults have a history of conduct disorder in childhood, and prospectively children with conduct disorder have an increased probability of antisocial behaviour in adulthood [5, 6]. Conduct problems are also associated with a range of other adverse outcomes throughout adult life, including relationship problems, mental disorder, physical health problems, substance abuse, and financial difficulties [7, 8] which accumulate to impose a large economic burden on society [9].

**Fig. 1:**
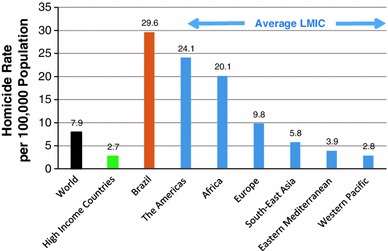
Homicide rates in world health organisation member states 2008. *LMIC* low and middle income countries. Source: World Health Organisation Global Burden of Disease. http://www.who.int/topics/global_burden_of_disease/en/. Accessed 26 Nov 2012

Most research on conduct problems has been conducted in high-income countries in North America, Western Europe and Australasia, and it is important to establish the extent of problems and underlying risk factors in other settings [10, 11]. Ninety per cent of the world’s 2.2 billion children and adolescents live in low- and middle-income (LMIC) countries [12], many of which are characterised by high levels of social and economic deprivation and violence. In this article, we review research on the prevalence of and risk factors for childhood conduct problems in Brazil, Latin America’s largest country, with one of the highest homicide rates in the world (see Fig. 1).

Brazil has the fifth largest population in the world: 197 million people [13], 30 % of whom are under age 18 [12]. Although Brazil’s gross national income is not low (US$11,500 per capita in 2011 [14]), it has persistently had one of the highest rates of inequality in the world: its 2012 GINI index (51.9) was the 16th highest out of 136 countries worldwide (the United States ranks 42nd, and the United Kingdom ranks 91st) [15]. In 2009, the poorest fifth of the population received just 2.9 % of the nation’s income compared with 58.6 % received by the richest fifth [16]. In the same year, 10.9 % of the nation’s population was poor (living on less than $2 per day [16]). Nevertheless, there have been considerable improvements in health outcomes in the Brazilian population in recent decades: between 1975 and 2007 infant mortality decreased from 114 to 19 (per 1,000 live births), and life expectancy increased from 52 to 73 years between 1975 and 2008 [17]. Access to education also increased substantially: the proportion of people with seven or more years of formal education increased from 19 to 47 % between 1976 and 2008 [17]. However, ranking 85th out of 187 countries on the Human Development Index in 2011 (this index combines indicators of life expectancy, educational attainment and national income [18]), Brazil still has considerable challenges in meeting the whole population’s needs for health care, education, and income. Moreover, violence has grown into a major public health problem in Brazil. Between 1980 and 2010, the rate of youth homicide (deaths by aggression among people under 20 years old) increased by 346 % [19]. In 2007, 12.5 % of all deaths were caused by violence, most of which occurred among young men [20].

One might expect a relatively high prevalence of childhood conduct problems in Brazil for two reasons: (1) rates of youth violence are high and violence is typically preceded by childhood conduct problems; (2) Brazilian children living in impoverished urban environments are likely to be exposed to multiple risk factors for conduct problems [21]. The argument that elevated conduct problems in Brazil are probable given high levels of risk exposure assumes that risk factors identified in other countries, such as low family income and exposure to violence, are also implicated in the development of conduct problems in Brazil. This needs empirical demonstration. Empirical studies are also needed in Brazil because there may be other risk factors, or combinations of risk factors there that have not been identified elsewhere, because nearly all major research programmes on antisocial behaviour have been conducted in high income countries [22]. To consider these issues and highlight future research needs, we conducted a systematic review of epidemiological studies of the prevalence of and risk factors for conduct problems in Brazil. No similar review has been conducted to date.

## Methods

### Systematic review of literature

We systematically searched for population-based studies reporting the prevalence of childhood conduct problems or associations between risk factors and childhood conduct problems, in Brazil. To be eligible for the review, the study must have met all the following criteria:

1. The study used random sampling, stratified probability sampling, or total sampling of children in schools, households, maternity hospitals or public health programmes. Samples recruited entirely from an institutionalised setting, for example a drug addiction centre, were excluded.
2. At least 100 children (≤18 years old) were assessed for conduct problems, conduct disorder or oppositional defiant disorder.
3. The study used a standardised measure of conduct problems, oppositional defiant disorder or conduct disorder, e.g. the Child Behavior Checklist [23, 24], the Strengths and Difficulties Questionnaire [25], or the Development and Wellbeing Assessment [26].
4. The study reported either the prevalence of conduct problems, oppositional defiant disorder or conduct disorder, or the association between at least one risk factor and at least one of these problems.

Published and unpublished studies were eligible. Studies could be reported in English or in Portuguese. Where we were aware of studies with relevant but unpublished results, we contacted directors of the studies and requested the results to include in the review.

**Fig. 2:**
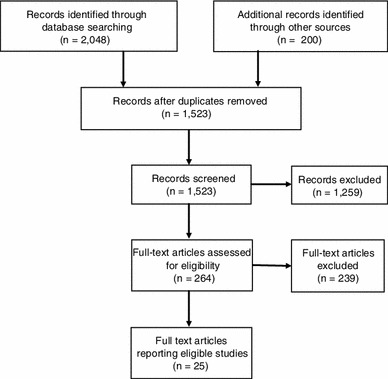
Flowchart of screening process to identify studies for the review

We searched the following electronic databases for eligible studies in May 2012: Social Science Citation Index, PubMed, and LILACS (a major index of scientific and technical literature of Latin America and the Caribbean). The following keywords were used (and they were also translated and entered into LILACS separately in Portuguese): [ODD OR oppositional defiant disorder OR CD OR conduct disorder OR conduct problems OR externalising OR crime OR violence OR delinquency OR illicit drug* OR substance use OR substance abuse] AND [cohort OR longitudinal OR prospective OR cross-sectional OR case control OR population] AND Brazil AND [prevalence OR rate OR incidence OR frequency OR risk factor]. To the list of references retrieved from electronic searches, we also added documents from our own archives, colleagues’ recommendations, and relevant articles in reference lists of retrieved reports. A flow chart of the search and screening process is shown in Fig. 2.

Two researchers [JM and EG] independently assessed the full texts for eligibility. On first assessment there was 88 % agreement on the studies that should be included or excluded. Remaining studies were re-examined and discussed to agree on inclusion or exclusion.

### Synthesis of evidence and meta-analysis

We conducted a meta-analysis of the prevalence of conduct problems using the inverse variance weight approach in random effects models. The effect size used in the analysis was the proportion of children with conduct problems. Confidence intervals were estimated based on the effect size and the number of participants in the study [27]. Weighted average effect sizes were calculated separately for studies that assessed conduct disorder and oppositional defiant disorder, and for studies that assessed conduct problems using screening instruments. For studies using screening instruments, among which there was significant variability in the results, we examined the following variables as possible moderators of effect sizes: the region of Brazil in which the study was conducted, whether recruitment of participants took place in schools or the community (referring to households, maternity hospitals, or public health programmes), response rates, the proportion of the sample that was male, informants on child behaviours, instruments used, and whether an impairment criterion was used to define cases of children with conduct problems. We also meta-analysed the association between child sex and child conduct problems. The meta-analysis was conducted using Comprehensive Meta-Analysis version 2.2.057.

We report the strength of association between risk factors and conduct problems using odds ratios, wherever possible. The odds of having conduct problems in a group of children are equal to the number of children with conduct problems divided by the number of children without conduct problems. The odds ratio equals the odds for children exposed to a risk factor divided by the odds for children not exposed to the risk factor. Thus the odds ratio (OR) represents how more or less likely conduct problems are among children with a risk factor compared with children without the risk factor. An OR >1.0 shows an increased probability of risk, whereas an OR <1.0 shows a reduced probability of risk; an OR of 2.0 or greater indicates strong association [28]. If studies did not report odds ratios, wherever possible we calculated them and their confidence intervals based on 2 × 2 tables.

We did not meta-analyse results on risk factors (except for child sex), because there were few studies that examined the same risk factors, and the methods used to measure and analyse risk factors often varied substantially between studies. Instead, we summarise the individual findings on risk factors from each study, and consider patterns across the studies in the Discussion section.

## Results

### Prevalence of childhood conduct problems in Brazil

Sixteen studies eligible for the review reported the prevalence of childhood conduct problems. Four of the studies used a diagnostic assessment tool (DAWBA: Development and Wellbeing Assessment) to estimate rates of conduct disorder and oppositional defiant disorder. These studies are summarised in Table 1. Rates of conduct disorder ranged from 0.6 to 2.2 %, and had a weighted average of 1.4 % (95 % CI 0.5–3.6). Rates of oppositional defiant disorder ranged from 2.0 to 3.2 % and had a weighted average of 2.4 % (95 % CI 1.7–3.5). Rates of any CD or ODD ranged from 2.6 to 7.0 % and had a weighted average of 4.1 % (95 % CI 2.1–7.9). Because there were few studies with results using diagnostic instruments, we did not analyse moderators that might explain variation in their results.

**Table 1:** Population-based studies reporting prevalence of childhood conduct disorder (CD) and oppositional defiant disorder (ODD) in Brazil

Study	Location	Sample	Number of participants (response rate)	Age range	% Male	Informants	Measure	CD/ODD prevalence
Anselmi et al. [68]	Urban: Pelotas, RS	All children born in maternity hospitals in 1993	4,448 screening (85 %) 280 diagnostic interview (95 %)	11–12	50	Parent and adolescent combined in clinical assessment	Diagnostic interview (DAWBA) after screening questionnaire (SDQ with impact)	CD 2.2 %
								ODD 2.1 %
								CD/ODD 4.4 %
Barros et al. [30]*	Urban: Pelotas, RS	All children born in maternity hospitals in 2004	3,585 (90.2 %)	7	51	Parent	Diagnostic interview (DAWBA)	CD 0.6 %
								ODD 2.0 %
								CD/ODD 2.6 %
Fleitlich-Bilyk and Goodman [37]	Urban/Rural: Taubaté, SP	Random sample of students in stratified sample of public/private schools	1,251 (83 %)	7–14	53	Parent, adolescent and teacher combined in clinical assessment	Diagnostic interview (DAWBA)	CD 2.2 %
								ODD 3.2 %
								CD/ODD 7.0 %
Goodman et al. [56]	Mainly rural: Ilha de Maré, BA	Random sample of students within random sample of schools	430 screening (100 %) 100 diagnostic interview (100 %)	7–14	50	Parent, adolescent and teacher combined in clinical assessment	Diagnostic interview (DAWBA) after screening questionnaire (SDQ with impact)	CD/ODD 3.4 %

DAWBA diagnosis using DSM-IV criteria. CD/ODD prevalence can be higher than CD and ODD combined because it includes other conduct disorders not otherwise categorised

Age in years. *SDQ* Strengths and Difficulties Questionnaire, *DAWBA* Development and Wellbeing Assessment

* Additional unpublished information provided by authors

Fourteen studies (summarised in Table 2) reported the prevalence of children with conduct problems using screening questionnaires. (Two of the studies using screening questionnaires also used diagnostic instruments to estimate CD and ODD, as shown in Table 1.) Eight of the screening studies used the Strengths and Difficulties Questionnaire (SDQ); four studies used questionnaires from the Achenbach System of Empirically Based Assessment (the Child Behavior Checklist, Teacher’s Report Form or Youth Self-Report); and one study used the Mini-International Neuropsychiatric Interview (MINI).^1^ The prevalence of conduct problems estimated using screening questionnaires ranged from 6.5 to 48.8 %. The weighted average was 20.8 % (95 % CI 15.9–26.9), but there was significant heterogeneity in the results (*Q* = 577.5, df = 13, *p* < 0.001). Given this heterogeneity, we examined moderating variables that might explain the variation.

**Table 2:** Population-based studies reporting prevalence of childhood conduct problems (CP) using screening questionnaires in Brazil

Study	Location	Sample	Number of participants (response rate)	Age range	% Male	Informants	Measure (CP case definition)	CP prevalence (%)
Anselmi et al. [39]^a^	Urban: Pelotas, RS	All children born in maternity hospitals in 1993 (half of sub-sample studied at 4 years)	634 (87 %)	4	50	Parent	CBCL (borderline-abnormal score)	31.8
Barros et al. [30]^b, *^	Urban: Pelotas, RS	All children born in maternity hospitals in 2004	3,750 (89 %)	4	52	Parent	CBCL (abnormal score)	21.9
Bordin et al. [31]	Urban: Embu, SP	Random sample of children in low-income households within random sample of census units	480 (81 %)	6–17	49	Parent	CBCL (abnormal score)	17.7
Cid [42]	Urban: São Carlos, SP	Random sample of children in 5 out of 8 selected public schools	321 (85 %)	6–10	49	Parent	SDQ (abnormal score)	39.3
Cruzeiro et al. [29]	Urban: Pelotas, RS	All adolescents in selected housing blocks in 79 census districts	1,145 (90 %)	11–15	48	Adolescent	MINI (≥2 symptoms)	29.2
Cucchiaro and Dalgalarrondo [33]	Urban: Campinas, SP	All students in random sample of public school classes	765 (77 %)	10–16	52	Adolescent and teacher combined with OR rule	SDQ (abnormal score with impairment)	6.5
Cury and Golfeto [34]	Urban: Ribeirão Preto, SP	Students in one public school (selection method not reported)	107 (75 %)	6–11	63	Parent and teacher combined with OR rule	SDQ (abnormal score with impairment)	9.8
Ferriolli et al. [43]	Urban: Ribeirão Preto, SP	Children from families enrolled in a public health programme	100 (79 %)	6–12	48	Parent	SDQ (abnormal score)	25
Lyra et al. [69]	Urban: São Gonçalo, RJ	Random sample of students within random sample of classes in random sample of public schools	372 (74 %)	7–11+	49	Teacher	TRF (borderline-abnormal score)	12.6
Paula et al. [70]	Urban: Barretos, SP	Random sample of students in public and private schools	327 (93 %)	11–15	43	Adolescent	SDQ (abnormal score with impairment)	8.6
Rodriguez et al. [44]	Urban: São Luís, MA	Random sample of singletons born in public and private maternity hospitals in 1997/98	805 (68 %)	7–9	52	Parent	SDQ (abnormal score)	48.8
Sherman et al. [35]	Urban: Salvador, BA	All students in one public school	344 (49 %)	11-18	38	Adolescent	YSR + Impact section of SDQ (borderline-abnormal score with impairment)	11.6
Silva et al. [71]^*^	Urban: Riberão Preto, SP	All singletons born in 10 maternity hospitals during 4 months in 1994	784 (68 %)	9–11	51	Parent	SDQ (abnormal score)	35.3
Vitolo et al. [36]^c^	Urban/Rural: Taubaté, SP	Random sample of students within stratified random sample of public/private schools	454 (83 %)	7–11	52	Parent	SDQ (borderline-abnormal score)	23.6

Age in years. *SDQ* Strengths and Difficulties Questionnaire, *CBCL* Child Behavior Checklist, *TRF* Teacher Report Form, *YSR* Youth Self Report, *MINI* Mini International Neuropsychiatric Interview. For CBCL/TRF/YSR, conduct problems = externalising sub-scale, abnormal score = clinical score

^a^Same sample as Anselmi et al. [68] in Table 1

^b^Same sample as Barros et al. [30] in Table 1

^c^Same sample as Fleitlich-Bilyk and Goodman [37] in Table 1

^*^Additional unpublished information provided by authors

Results of analyses of moderator variables that were measured as categories are shown in Table 3. Significantly higher rates of conduct problems were found in studies that recruited children from the community (in household surveys, maternity hospitals in birth cohort studies, or public health programmes) compared with studies recruiting from schools. Higher rates of conduct problems were reported by parents, followed by children, then teachers, and lowest rates were found in two studies using multiple informants—which also used an impairment criterion to identify children with probable conduct disorder. Considering the assessment instrument used, the highest rate of conduct problems was reported in the single study that used the MINI questionnaire, with lower rates being reported in studies using the Strengths and Difficulties Questionnaire and Achenbach scales. A much lower prevalence of conduct problems was found among studies using an impairment criterion to identify children with conduct problems compared with studies using symptom scores only. There were no significant differences in the results according to the region of Brazil in which the study was conducted.

**Table 3:** Moderators explaining variance in the prevalence of conduct problems in studies using screening instruments

Moderator variable	Number of studies	Prevalence (95 % CI)	*QB*	*p*
Region of Brazil			3.2	0.2
Northeast	2	26.2 % (4.8–71.3)		
Southeast	9	17.6 % (11.2–26.5)		
South	3	27.3 % (21.3–34.3)		
Recruitment location			5.9	<0.05
Schools	7	13.9 % (7.7–23.8)		
Community	7	29.3 % (22.1–37.7)		
Informants			37.5	<0.001
Parent	8	29.7 % (22.4–38.2)		
Child	3	14.8 % (6.0–32.3)		
Teacher	1	12.6 % (9.6–16.4)		
Multiple	2	7.3 % (5.1–10.5)		
Instrument			10.2	<0.01
SDQ	8	21.3 % (12.8–33.4)		
Achenbach scales	5	18.5 % (13.5–24.7)		
MINI	1	29.2 % (26.6–31.9)		
Impairment required			35.5	<0.001
Yes	4	8.8 % (6.5–11.7)		
No	10	27.6 % (21.7–34.4)		

We used meta-regression to examine whether variation in study results was associated with two study characteristics measured at the interval-level: the study response rate and age of the study children (defined as the mid-point of the age range in years). Studies with higher response rates had smaller effect sizes (a lower proportion of children with conduct problems; *B* = 0.012, *p* < 0.001). Child age was not significantly associated with study results.

### Risk factors for childhood conduct problems in Brazil

Twelve studies examined associations between individual, family or social risk factors and children’s conduct problems in Brazil. The only variable which was analysed frequently enough to justify a meta-analysis was child sex. For all other variables, we summarise findings from each study separately, reporting odds ratios and confidence intervals wherever possible for significant (*p* < 0.05) associations, and listing any non-significant results.

Results on sex differences were inconsistent across seven studies^2^ that used screening questionnaires: three found significantly higher rates of conduct problems among boys, two found significantly higher rates among girls, and two studies found no significant sex difference. Meta-analysing these screening studies, the weighted average odds ratio (comparing boys to girls) was not significant (OR = 1.2; 95 % CI 0.9–1.7), and heterogeneity in the results was significant (*Q* = 25.5, *p* < 0.001). In the only study [37] that compared rates of CD/ODD between boys and girls, there was a significantly higher rate among boys (OR = 3.0; 95 % CI 1.8–5.0).

Brion et al. [38] examined perinatal risk factors for conduct problems at age 4 years among 523 children in the Pelotas Birth Cohort Study 1993 (see the study by Anselmi et al. [39] in Table 2). Pelotas is a city in the state of Rio Grande do Sul (RS) in the southern region of Brazil. The analyses were focused on effects of maternal smoking in pregnancy on children’s mental health. Higher conduct problem scores were associated with maternal smoking in pregnancy (OR = 1.7; 95 % CI 1.2–2.4) and with maternal psychiatric problems (OR = 3.1; 95 % CI 2.1–4.5). However, conduct problems were not significantly associated with paternal smoking during the mother’s pregnancy, maternal and paternal education, family income, or social class. Maternal smoking in pregnancy remained significantly predictive of conduct problems at age 4 after controlling for all other variables.

Using data on 4,423 participants in the same Birth Cohort Study in Pelotas (RS), Anselmi et al. [40] examined perinatal and age 11 risk factors for conduct problems at age 15 (on the Strengths and Difficulties Questionnaire), focusing on the effects of family income change between birth and the age of 11 years. Higher conduct problem scores were associated with non-white skin colour, maternal smoking during pregnancy, young maternal age, and low maternal schooling in the perinatal assessment; family income change from birth to age 11; and stressful life events, poor maternal mental health, and the mother living without a partner when the child was aged 11. Controlling for all other variables, family income change was significantly predictive of conduct problem scores at age 15. Compared with children with high family incomes at both birth and age 11 (high–high), children in the following income groups had higher conduct problem scores at age 15: high–low, intermediate–intermediate, intermediate–low, low–intermediate, and low–low.

Caputo and Bordin [41] conducted a case–control study comparing rates of conduct problems (clinical level problems on the Youth Self-Report questionnaire) between 207 primiparous pregnant adolescents and 308 sexually active but never-pregnant adolescent girls (13–17 years old) in Marília (SP). Conduct problems were less likely among pregnant girls (13.0 %) than among non-pregnant girls (20.8 %), equivalent to a significant odds ratio of 0.6 (95 % CI 0.4–0.9).

Cid [42] compared levels of conduct problems by various parenting and family characteristics in a study of 321 elementary school children (6–10 years old) in São Carlos (SP) (see Table 2). Higher conduct problem scores were associated with receiving inconsistent discipline, relaxed discipline, physical abuse, lower levels of positive parenting, poor parent–child communication, the child having comorbid mental health problems, parental mental health problems, not living with both parents, fighting within the family, and repeating a school grade. Conduct problems were not significantly associated with positive parental monitoring, negative parental monitoring, moral parenting, negligent parenting, having clear family rules and responsibilities, or school performance.

Cruzeiro et al. [29] investigated risk factors for conduct problems among 1,145 adolescents (11–15 years old) in a cross-sectional household survey in Pelotas (RS) (see Table 2). The following risk factors predicted higher conduct problem scores: higher age (13–15 compared with age 11–12 years), low social class (OR = 1.7; 95 % CI 1.1–2.5), having repeated school years (OR = 1.5 for twice or more; 95 % CI 1.1–2.1), no religion (OR = 1.3; 95 % CI 1.0–1.6), no participation in protestant service or catholic mass (OR = 1.4; 95 % CI 1.1–1.8), alcohol use/drunkenness (OR = 2.6; 95 % CI 2.0–3.5 and OR = 3.0; 95 % CI 1.8–5.1, respectively), smoking cigarettes (OR = 2.3; 95 % CI 1.3–3.9), drug use (OR = 5.9; 95 % CI 3.3–10.6), depression (OR = 5.1; 95 % CI 1.1–25.2), and victim of bullying (OR = 2.1; 95 % CI 1.6–3.0). Years of schooling were not significantly associated with conduct problem scores. In a multivariate model, significant independent predictors were male sex, age, lower socioeconomic status, use of alcohol or drugs, and victim of bullying.

Cucchiaro and Dalgalarrondo [33] examined whether school students in a poor, outer region of Campinas (SP) had different rates of conduct problems compared with students in the central area of the city, in a cross-sectional study of 424 children (10–13 years old; see Table 2). The outer region of the city was characterised by lower levels of paternal educational, lower indices of wealth, and a higher proportion of black children. Rates of conduct problems were similar between children in the two areas (6.0 % in central areas and 7.9 % in outer-city areas; OR = 1.3; 95 % CI 0.8–2.4; not significant).

Curto and colleagues [32] examined risk factors for conduct problems among 248 adolescents (11–17 years old) in a cross-sectional study in Embu (SP) (see Bordin et al. [31] in Table 2). Risk factors associated with higher levels of conduct problems were severe physical punishment (OR = 2.8; 95 % CI 1.4–5.8), adolescent internalising problems (OR = 7.8; 95 % CI 3.3–15.7), and maternal anxiety/depression (OR = 2.9; 95 % CI 1.5–5.6). Variables not significantly associated with conduct problems in bivariate tests were adolescent age, maternal education, maternal paid work, marital violence, father absence, and socioeconomic status. In a multivariate model, significant risk factors for conduct problems were severe physical punishment, internalising problems, father absence, and three interactions: age*internalising, age*maternal anxiety/depression, and maternal work*socioeconomic status. The three interactions showed that (1) younger adolescents had higher risk for conduct problems than older adolescents only if they also had internalising problems; (2) older adolescents had higher risk for conduct problems than younger adolescents only if their mother was anxious/depressed; and (3) adolescents with non-working mothers had higher risk for conduct problems than other adolescents only in situations of low socioeconomic status.

Ferriolli et al. [43] assessed risk factors for conduct problems in a cross-sectional study of 100 children (6–12 years old) in Riberão Preto (SP) (see Table 2). The only variables significantly associated with conduct problems were not having a well-defined daily routine and not having a place to do homework in the house. Non-significant variables were poor parental relations, maternal depression, maternal stress, leisure activities, financial instability, and socioeconomic level.

Rodriguez et al. [44] examined associations between perinatal and socioeconomic factors measured in the first year of life and conduct problems at ages 7–9 years in the São Luís (MA) Prospective Birth Cohort Study (see Table 2). Prevalence ratios (PR) were used to report the relative probability of conduct problems comparing children with and without risk factors. Conduct problems were more common among children whose mothers had 5–8 years of schooling compared with over 8 years of schooling (PR = 1.4; 95 % CI 1.2–1.7), and among children in middle income families compared with children in families with high income (PR = 1.3; 95 % CI 1.1–1.6). Nonsignificant variables were preterm birth, birth weight, maternal/paternal age, and mother’s marital status. In a multivariate model, the only significant predictors were male sex and lower maternal schooling.

Sherman et al. [35] conducted a cross-sectional study of 263 adolescents (11−18 years old) in Salvador (BA) (see Table 2). Risk factors for conduct problems were analysed separately for boys and girls. For boys, conduct problems were associated with low religiosity (OR = 1.3; 95 % CI 1.0–1.6), low family cohesion (OR = 2.0; 95 % CI 1.2–3.5), and family conflict (OR = 2.5; 95 % CI 1.4–4.4). For girls, conduct problems were associated with parents not being married (OR = 5.9; 95 % CI 1.7–20.2), low family cohesion (OR = 2.1; 95 % CI 1.5–3.0), and family conflict (OR = 2.5; 95 % CI 1.7–3.8). Variables not significant for either sex were race, maternal/paternal education, and parental unemployment. In multivariate models, the only significant correlates of conduct problems were low family cohesion and family conflict for girls.

Vitolo et al. [36] assessed risk factors for conduct problems among 454 children (7–11 years old) in a cross-sectional study in Taubaté (SP) (see Table 2). Children being hit with a belt was associated with increased risk for conduct problems (OR = 2.2; 95 % CI 1.2–2.3), as was parental mental health problems (OR = 2.1; 95 % CI 1.4–3.3) and low social class (OR = 1.6; 95 % CI 1.1–2.3). All these risk factors remained significant in multivariate models. Goodman et al. [45] also examined correlates of conduct problems among 1,112 children in the same study (including children with a wider age range 7–14 years old). Risk factors independently associated with conduct problem symptoms in a multivariate model were not living with both biological parents, alcohol abuse in the family, parental stress, and harsh physical punishment. Non-significant variables were child age, general health, and IQ.

## Discussion

In a systematic review of the literature, we found 16 population-based studies reporting the prevalence of childhood conduct problems and nine studies examining risk factors for conduct problems in Brazil. Findings from these studies are discussed in relation to the international literature.

### Prevalence of conduct problems

Fourteen Brazilian studies that assessed conduct problems using screening questionnaires had an average prevalence rate of 20.8 %. However, there was significant variability in their results. This variability was associated with participant recruitment location, response rates, study informants, assessment instruments, and use of impairment criteria to define cases, emphasising the importance of methodological considerations in interpreting study results. This was also the main conclusion drawn by Canino et al. [46] in their review of prevalence studies of CD and ODD, in which only methodological features, and not geographical location of the studies, explained variation in results.

In our review of Brazilian studies, the screening instrument most widely used was the Strengths and Difficulties Questionnaire (SDQ). The average prevalence of conduct problems assessed on the SDQ was 21.3 % in eight Brazilian studies including 3,663 children 6–16 years old. The two largest studies outside of Brazil that have measured conduct problems using the SDQ come from Britain and the United States [47]. In the British study of 10,298 children aged 5–15, the proportion of children with conduct problems^3^ was 12.7 %. Among 9,878 American children aged 4–17, the proportion was 10.7 %. Thus, children in the Brazilian studies in this review had roughly double the rate of conduct problems assessed on the SDQ, compared with British and American children.

In the four Brazilian studies that used a diagnostic instrument (DAWBA), average rates of disorder among 7–14 year olds were 1.4 % for CD, 2.4 % for ODD, and 4.1 % for any CD or ODD. The particular instrument used (DAWBA) provides conservative estimates of disorder compared to other instruments such as CAPA or DISC [48], emphasising the importance of comparing results from Brazil to studies in other countries that used the same instrument. Studies using DAWBA in other countries found the following rates of CD and ODD, respectively: 1.5 and 2.3 % among 5–15 year olds in Britain [2]; 0.5 and 2.5 % among 8–10 year olds in Norway [49]; 2.9 and 5.9 % among 5–10-year-olds in Bangladesh [50]; and 1.8 and 4.0 % among 7–10-year-olds in Yemen [51]. The prevalence of any CD or ODD was 8.6 % among in 7–14-year-olds in Russia [52]. Thus, rates of CD and ODD in Brazilian studies using DAWBA were generally similar or lower than those found in several other countries.

### Different conclusions based on screening questionnaires and diagnostic instruments

Why were conduct problems in Brazil more prevalent than in other countries when defined using questionnaire symptoms, but similar or lower when assessed in terms of CD and ODD? It is possible that Brazilian children genuinely have higher levels of antisocial behaviour even though they do not meet diagnostic criteria for CD/ODD. For example, Brazilian children might learn aggressive behaviours to adapt in violent environments or in response to peer pressure among antisocial groups, without this causing social or educational impairment—required for diagnosing CD and ODD. Consistent with this hypothesis, Brazilian questionnaire studies defining conduct problems using an impairment criterion showed significantly lower rates of problems than studies examining only behavioural symptoms [see also 53].

An alternative explanation for the unusually high rate of conduct problems found in Brazilian questionnaire studies is informant bias: Brazilian parents might have a lower threshold for reporting child problem behaviours than parents elsewhere. Brazilian parents with lower literacy levels might be less likely to deliberate about fine distinctions or qualifications on questionnaire items and therefore might over-report serious problems. For example, they might be more likely to choose an option “definitely true” rather than “somewhat true” about the presence of a particular symptom, producing artifactually higher child behaviour scores in Brazil compared with other countries [for discussions, see 54–56]. Consistent with this possibility, three Brazilian studies using multiple or teacher informants found lower rates of child conduct problems compared with studies based only on parent reports. Given the theoretical plausibility of both interpretations of the high rate of conduct problems on screening questionnaires in Brazil (that this reflects either genuinely high levels of antisocial behaviour or reflects informant bias), methodological studies including multiple types of instrument in the same study are required to resolve the issue.

Another question raised by our results is why there are such high levels of serious violence in Brazil if childhood conduct disorder is not more common than in high-income countries. One possible answer is that sub-clinical childhood behavioural problems (which seem higher in Brazil) gradually develop into more serious criminal and violent behaviour in the context of inequality, poverty, low quality public schools, lack of job opportunities for poor and low-educated youth, organised crime and an under effective criminal justice system. As Eisner [57] has argued, “the specific configuration of problem behavior in a country may… depend on the way in which the transition to early adulthood is molded by the concurrence of opportunities and lifestyles related to general affluence, the intensity of informal social control associated with different family and household patterns, and the strain originating from the degree to which life chances and resources are unequally distributed in a society.”

Longitudinal studies are needed in Brazil to investigate these issues, to identify how antisocial behaviour develops from childhood to adulthood, and to specify the risk processes leading to childhood conduct problems and later crime. However, even basic epidemiological evidence on variation in antisocial behaviour by age is lacking in Brazil. Thus, detailed cross-sectional data on age patterns in conduct problems (as are available elsewhere [58]) would fill a critical gap in the knowledge base in Brazil.

### Risk factors for conduct problems

Generally, risk factors for conduct problems identified in Brazilian studies were very similar to those found in the international literature. Risk factors that were replicated in at least two of the ten Brazilian studies reviewed were comorbid child mental health problems, educational failure (repeating a school year), low religiosity, harsh physical punishment and abuse, parental mental health problems, single-parent family, and low family socioeconomic status.^4^ All these are well-established risk factors for antisocial behaviour in other countries [22, 59, 60]. Given the possibility of contextual differences in risk factor effects [61], it is important that most risk factors examined were replicated in Brazilian samples.

Given the almost universal finding that antisocial behaviour is more common among males than females [62], it was surprising that Brazilian studies were somewhat mixed on sex differences. Although one study found higher rates of CD/ODD among boys than girls, our meta-analysis of screening studies revealed no significant sex difference in rates of conduct problems. A possible explanation is that, in contexts of extreme poverty and violence like Brazil, environmental influences swamp the effects of other individual/biological factors that cause sex differences in other contexts. To investigate this issue, it would be desirable to compare exposure to and effects of risk factors between boys and girls through time in longitudinal studies in Brazil.

A critical issue for research is identifying which risk factors actually cause increases in childhood conduct problems, as opposed to merely mark genetic effects or other environmental risk mechanisms [63, 64]. Several Brazilian studies used regression models to statistically control for confounding factors, but we found no research using other methods to investigate causal risk effects. There is a need for new research to establish which risk factors actually cause conduct problems in Brazil, including studies with genetically sensitive designs (e.g. twin studies), natural experiments, propensity score matching, and analyses of within-individual change through time [63–65]. Risk mechanisms should also be investigated in Brazil that have not been extensively studied in high-income countries, for example effects of malnutrition [66]. In a prospective cohort study in Mauritius, malnutrition at the age of 3 years was predictive of conduct problems at ages 11 and 18 years independently of psychosocial adversity [67]. No similar studies were found in Brazil, and future research should include this type of risk factor, which is more common than in high-income countries and is relatively understudied.

### Limitations and conclusions

Our review was of course limited by the available primary evidence. Without a larger number of studies to draw on, we could not conduct multivariate meta-regression analyses to identify the most important characteristics that explained variation in study results. The studies we did identify for this review focused almost exclusively on psychosocial risks, and we were unable to consider biological mechanisms that may interact with environmental stress to cause conduct problems in Brazil.

We conclude that results on the prevalence of childhood conduct problems in Brazil are complex, with important variation according to study methodology. In particular, there is a higher prevalence of conduct problems in Brazil compared with other countries when screening questionnaires are used, but similar levels of conduct disorder and oppositional defiant disorder when diagnostic instruments are used. Research on risk factors in Brazil has mainly replicated findings from high-income countries concerning individual characteristics, family processes, and social contexts. The next generation of research in Brazil should use longitudinal designs to identify developmental processes and causal risk mechanisms for childhood conduct problems and later antisocial behaviour.

## Footnotes

1 We classify MINI as a screening instrument in the study that applied it [29] because the definition of conduct problems used in that study was “two or more behavioural symptoms”, which does not correspond to diagnostic criteria of CD or ODD. Among studies using the SDQ, one applied the “borderline” cut-point and seven studies applied the “abnormal” cut-point (as defined at www.sdqinfo.com). Among studies using Achenbach questionnaires, three applied the “borderline” cut-point to define children with high conduct problem scores, and two applied the “clinical” cut-point. We refer to this “clinical” cut-point as “abnormal” in Table 2, for consistency with the SDQ. Three studies using the SDQ and one using an Achenbach questionnaire also required the presence of impairment symptoms for children to be classified as having conduct problems.

2 These were Barros et al. [30], Bordin et al. [31], reported in Curto et al. [32], Cruzeiro et al. [29], Cuccihiaro and Dalgalarrondo [33], Cury [34], Sherman et al. [35], and Vitolo et al. [36].

3 Scoring in the clinical range (4–10) as reported by parents, without requirement of impairment. This was the method and definition used most frequently in the Brazilian studies.

4 Although several studies did not find a significant association for low socioeconomic status and conduct problems, this might have been because of lack of power or homogeneity of samples in socioeconomic position. Certainly, no study found a significant reverse association in the opposite direction, i.e. low socioeconomic status being associated with reduced probability of conduct problems.
